# Maltreated and misdiagnosed finger fractures: a malpractice claim analysis from the Swedish national patient insurance register 2011–2021

**DOI:** 10.1016/j.jham.2025.100382

**Published:** 2025-11-12

**Authors:** Jonny K. Andersson, Amanda Davidsson, Marcus Sagerfors

**Affiliations:** a Atleva, Gothenburg Handcenter, Gothenburg, Sweden; b Department of Orthopaedics, Institute of Clinical Sciences, The Sahlgrenska Academy, University of Gothenburg, Gothenburg, Sweden; c Department of Orthopedics and Hand Surgery, Faculty of Medicine and Health, Örebro University, SE, 70182 Örebro, Sweden

**Keywords:** Finger fractures, Maltreatment, Misdiagnosis, Complications, Costs

## Abstract

Finger fractures are among the most common fractures of the upper limb. The number and cost of maltreated and misdiagnosed finger fractures in Sweden is unknown. The aim was to study the number, complications, causes, and cost of maltreated and misdiagnosed finger fractures, 2011–2021, in Sweden.

Claims matching the prespecified ICD-10-SE codes S62.6–7 (finger fractures) and T92.2 (sequelae after finger fracture) during the 2011–2021 timeframe were identified. The data were extracted from the Swedish National Patient Insurance Company Register and analyzed in terms of epidemiology and cost.

Of the 1621 assessed cases, 384 reported maltreated and misdiagnosed finger fractures were found. The mean age was 41 years (range 2–88). Thirty-one percent of the healthcare-related injuries occurred in emergency care, primarily due to maltreatment leading to malunion after non-operative treatment. Thirty-six percent of healthcare-related injuries occurred in specialist departments, mostly due to malunion after surgical intervention. In primary care, the leading cause was misdiagnosis, often due to inadequate examination and lack of X-ray examination. The total aggregated direct and indirect costs amounted to SEK 25 557 200 (USD 2 505 608, Euro 2 165 864, Yen 424 249 520).

**Conclusion:**

Finger fractures affect people of all ages and can lead to significant socioeconomic and medical invalidity. Maltreated fractures mainly occur in emergency care (due to malunion after immobilization) and specialist care (due to malunion after surgery). Misdiagnosed fractures were more common in primary care. A suggestion for claims prevention would be improved physician education, updated guidelines regarding the use of X-rays, seeking specialist opinions in uncertain cases, evaluating surgical technique, and optimizing postoperative care could probably help reduce the number of these injuries.

## 1 Introduction

The hand is a complex structure with the ability for fine and gross motor skills.^1^ Fractures in the hand are among the most common injuries in the upper limbs, affecting 3.7 per 1000 men and 1.3 per 1000 women every year.^2^ One of the most common hand fractures is finger fractures.^3^^,^^4^ The treatment of finger fractures aims to provide adequate stability, ensure early appropriate range of motion, and minimize soft tissue damage. This can be achieved through simple splint immobilization or more complex invasive internal fixation.^5^

Most finger fractures are treated non-operatively, and usually fractures with an angular misalignment (<15°) and shortening (<5 mm) are accepted for such treatment. Rotational misalignments should never be accepted, and require further action.^6^ Some fractures are unstable and can require surgical fixation.^3^ Different types of fractures may lend themselves better or worse to specific surgical techniques, but it is often the surgeon's expertise and clinical judgment that determine the most appropriate method of surgical treatment.^7^ Physical therapy should start as soon as the fracture is clinically healed to prevent stiffness.

Missed fractures are among the most common diagnostic errors in the emergency department, due to misreading or not obtaining X-rays.^8^ The lack of a multidimensional view in X-rays can result in underestimation of the severity of the injury and lead to inadequate management. The most commonly missed fracture at the primary care level has been hand fractures.^9^ The long-term consequences of maltreated finger fractures vary between patients and depend on the type of fracture and location. The range of complications after finger fractures includes stiffness, malunion, arthritis, nonunion, infection, and chronic pain 10.

Löf is a Swedish national insurance company handling healthcare-related injuries. It is co-owned by the Swedish health care regions who provide care for Swedish citizens. Since 2000, over 200 000 claims have been filed, including those for maltreatment or misdiagnosis of hand fractures, or secondary injuries from such fractures (ICD codes S62.6, S62.7, T92.2). While the patient must file the claim, the healthcare provider is responsible for informing the patient of potential maltreatment and of how to submit a claim to Löf. An attorney is not needed to file a claim. When an injury is reported to Löf, it is responsible for investigating the claim. This involves gathering documents from the patient's records and having an independent expert physician analyze the information to determine the sequence of events, whether the injury or complication was avoidable, whether the patient is eligible for compensation, and, if so, what amount should be received.^11^ If the injury/claim is deemed not avoidable, the claim is rejected.

To our knowledge, no national studies have examined maltreated or misdiagnosed finger fractures in Swedish healthcare. Understanding the impact on patients’ quality of life, and the number, causes, and locations of these incidents, is essential to improve treatment.

The primary aim of the study was to investigate complications, of maltreated and misdiagnosed finger fractures reported to Löf between 2011 and 2021. Secondary aims of the study were to better understand the most common fractures, why they were maltreated or misdiagnosed, what trends can be seen over time, whether any surgical method was more likely to result in healthcare-related injuries, and what costs these injuries imposed on the individual and society. Furthermore, we aimed to analyze what measures can be taken to reduce the number of reported maltreatments.

## 2 Material and methods

### 2.1 Study design

The study is based on a register of data collected retrospectively from Löf. Relevant ICD-10-SE (International Classification of Disease, Tenth Revision, Swedish) diagnosis codes were selected (S62.6-7, T92.2). The study was approved by the Swedish ethical review authority No 2022-07279-01.

**Table 1:** Demographics for included cases, n (%).

	Male	Female	Total
**Sex**	160 (42 %)	224 (58 %)	384
**Age**			
0–17	37 (23 %)	18 (8 %)	55 (14 %)
18–24	20 (13 %)	22 (10 %)	42 (11 %)
25–44	53 (33 %)	64 (29 %)	117 (31 %)
45–64	42 (26 %)	84 (37 %)	126 (33 %)
>65	8 (5 %)	36 (16 %)	44 (11 %)
Average (SD)	34.4 (18.4)	45.3 (18.9)	40.8 (19.5)
Median (min-max)	31.5 (2–83)	47 (2–88)	41 (2–88)
**Profession**			
White-collar	32 (20 %)	64 (29 %)	96 (25 %)
Blue-collar	59 (38 %)	69 (31 %)	128 (33 %)
Non-employable	48 (31 %)	32 (14 %)	80 (21 %)
Retired	11 (7 %)	45 (20 %)	56 (15 %)
Umemployed	3 (2 %)	4 (2 %)	7 (2 %)
Unknown	4 (2 %)	10 (4 %)	14 (4 %)
**Deemed as avoidable injury**			
Yes	110 (69 %)	160 (72 %)	270 (70 %)
No	48 (30 %)	61 (27 %)	110 (29 %)
Not evaluated	1 (1 %)	3 (1 %)	4 (1 %)
Average PS% (SD)	2 (1.6)	2 (1.4)	2 (1.5)

Expressed in part sums, sums, percentages and described through standard deviation and range (min-max). Abbreviations: n = part sums. N = total. % = percentage of total. SD = standard deviation. Min = minimal value. Max = maximal value. PS% = degree of invalidity due to healthcare injury.

### 2.2 Data extraction

Pseudonymized data were extracted retrospectively from the register, via remote access on a virtual private network using double authentication. The data collected were based on set parameters, which include the following: level of care, primary diagnosis, type of fracture, treatment received, complications, age at injury, profession, year reported to Löf, eligibility for compensation, and amount of compensation. Personal data were not collected.

Type of profession was categorized as white collar, blue collar, unemployed, retired, or unemployable.^12^ Unemployable was defined as a person unable to be employed, either due to studies (being in school/university), age, or other circumstances other than unemployment or retirement. Filing a complaint to Löf can be done by the patient who experiences a healthcare-related injury the same day or up to 10 years after the injury. Compensation is granted if the healthcare related injury was deemed avoidable based on best practice at the time of injury. Medical invalidity is calculated based on a set framework. The patients can appeal in terms of denied complaints within 3 years. Appeals can be addressed either as a question of internal review to Löf or to the Patient Claims Panel (a group of physicians, representatives from the society, independent insurance company representatives and judge/lawyer, all appointed by the government). Only 10 % of all denied complaints to Löf are appealed and 10 % of them are changed.

### 2.3 Costs

Direct cost included the amount paid out for each insurance claim, excluding taxes, interest, and additional fees, as these are unrelated to the injury itself. The inflation rate was not considered. Indirect cost included sick leave and other work-related payouts. Due to an inconsistency in data reporting in Löf, an estimate was made based on the recommended duration of sick-leave for the injury according to the Social Services in Sweden, together with the average salary and national guidelines for sick pay.^13^

### 2.4 Data analysis: statistics

The Mann–Kendall test in SPSS was used to calculate the statistical trend over time as the dataset was relatively small and with a non-normal distribution. This test compares data points to earlier ones and does not assume any specific distribution, making it more suitable than linear regression for trend analysis over time. Statistical significance was set to *p* < 0.05. The trend was based on the year the injury was reported to Löf rather than the year when the injury occurred. Statistical figures were created using Excel, with categorical variables presented as counts and percentages, and continuous variables presented as median (range) and mean (SD) values.

The study obtained ethical approval from the Swedish Ethical Review Authority (Dnr 2022-07279-01). If a patient wishes to make a claim due to maltreatment to Löf, they give consent for the data to be used in research. At the time of filing, the patients have the option to deny use of their information in accordance with the opt-out principle.

## 3 Results

### 3.1 Demographics and epidemiology

**Fig. 1:**
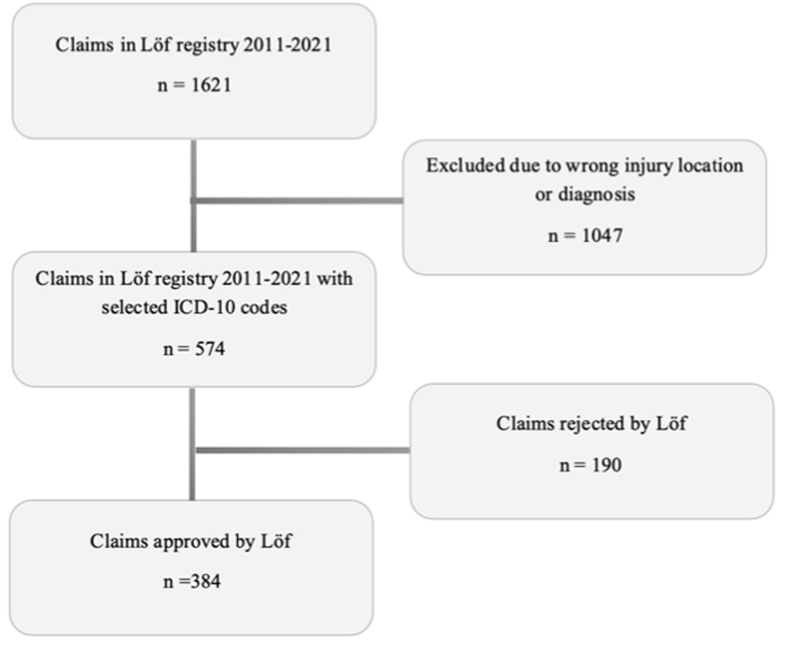
Search strategies. Selection of included cases from Löf registry and reasons for exclusion. Abbreviations: Löf = Swedish National Insurance Company; ICD10 = International Classification of Diseases, Tenth edition; n = part sum.

Between 1 January 2011 and 31 December 2021, 1621 cases of hand fractures were reported to Löf. Of these, 574 patients met the inclusion criteria (Fig. 1) and 384 (66 %) patients were compensated due to healthcare-related injury. Most of the patients were female (Table 1). Of the included cases, 99 % (*n* = 381) were granted compensation from Löf. The total paid-out compensation for all included cases amounted to SEK 11,092,400.

### 3.2 Trend

**Fig. 2:**
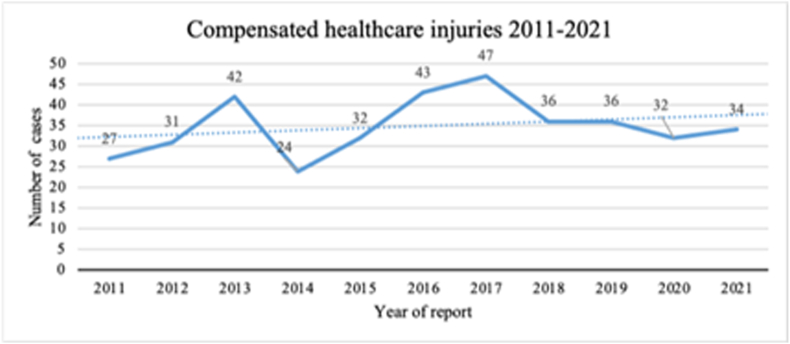
Number of accepted finger fracture patient injuries (claims) per year, 2011 to 2021 (year of report). Trend over time; p = 0.35 (Mann–Kendall test).

No significant trend was seen over time (p = 0.35), as displayed in Fig. 2.

### 3.3 Level of care

The emergency department was the most common level of care where healthcare-related injuries occurred, mostly due to maltreatment and malunion of the fracture after non-operative treatment with a plaster cast. The cases that were misdiagnosed at the emergency department (31 %, *n* = 119) were usually due to laceration injuries or animal bite injuries that were not X-rayed.

The specialist department was the second most common level of care where healthcare-related injuries occurred (36 %, *n* = 138) and were reported to and accepted by Löf. The most common healthcare-related injury was maltreatment (97 %, *n* = 134), often due to malunion of the fracture after surgical intervention.

Twenty-three percent (*n* = 90) of healthcare-related injuries occurred in primary care, mostly due to misdiagnosis, often from inadequate examination and lack of X-ray examination after trauma. Inadequate examination included cases in which patients were not X-rayed, were diagnosed with a distorsion, or were advised to stay home to see whether the pain subsided. Maltreated fractures in primary care were mainly caused by non-operative treatment, leading to malunion of the fracture.

**Fig. 3:**
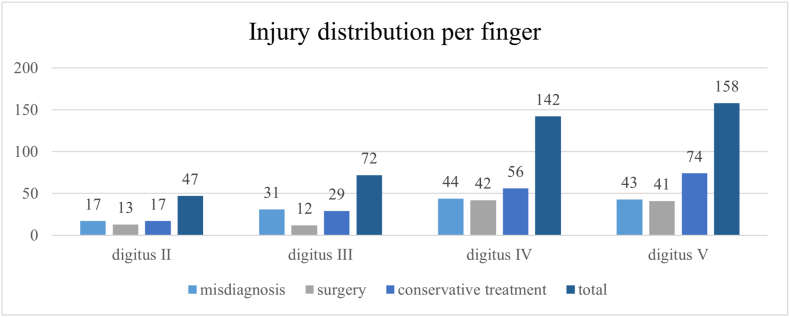
Injury distribution per finger, divided into three categories based on initial treatment.

Of the 384 cases approved for compensation, one per cent involved patients who were not injured at any of the three main injury locations. These patients were already hospitalized for another reason when they fell and sustained a fracture that was missed by staff. The most common finger fractured in healthcare-related injuries was the little finger, i.e., digitus V (Fig. 3).

**Fig. 4:**
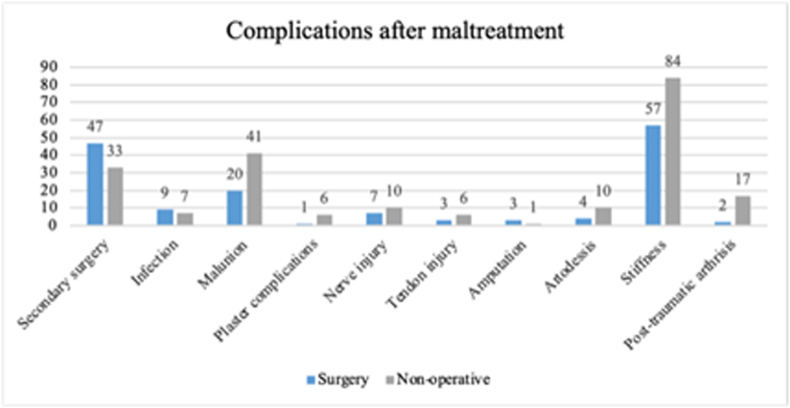
Number of complications (claims) after maltreated injuries divided by initial treatment, surgical intervention, or non-operative treatment.

Secondary surgery after initial treatment was higher in the group that received surgical intervention as a primary treatment. Stiffness was the leading complication for both groups regardless of initial treatment. Infection after initial treatment was similar in both groups: in the surgical intervention group, it was post-surgical infection; in the non-operative treatment, it was due to the nature of the injury, which often was an animal bite that led to fracture and a secondary infection. Malunion of a fracture that was not a candidate for secondary surgery was higher in the group receiving non-operative treatment (Fig. 4).

Out of 134 patients who were maltreated in the specialist department, 95 patients received surgical intervention. The most common surgical intervention was K-wire fixation (78 %, *n* = 76). Twelve patients (12 %) received fixation with screws, six plate fixation (6 %), three external fixation (3 %), and one fixation with osteo-suture (1 %). Two of the included patients had multiple fractures in the fingers and received multiple surgical techniques for that reason. The most common complications of surgical intervention with K-wire fixation were stiffness and re-operation. For the other surgical techniques, stiffness, re-operation, and malunion of the fracture were among the most common complications.

### 3.4 Analysis of cost

#### 3.4.1 Direct cost

Out of the 384 included cases, 99 % (*n* = 380) received compensation directly from Löf. The total amount of compensation for all patients was SEK 11,092,400 (USD 1 087 490, Euro 940 034, Yen 184 133 840) (SEK 4,835,223 (USD 474 042, Euro 409 465, Yen 80 264 702) for men and SEK 6,257,267 (USD 613 458, Euro 530 277, Yen 103 870 632) for women).

No other cases received compensation from an external insurance company.

#### 3.4.2 Indirect cost

The sick leave for finger fractures recommended by the Swedish social services varied based on the profession of the patient. It ranged from three weeks for white-collar work to three months for blue-collar professions.

The average monthly salary in Sweden before tax was SEK 39 900 (USD 3912, Euro 3381, Yen 662 340, with men earning SEK 42 000 (USD 4118, Euro 3559, Yen 697 200) and women SEK 37 800 (USD 3706, Euro 3203, Yen 627 480) (as of November 2023). When on sick leave, patients received about 80 % of their monthly salary, compensated by both the Swedish Social Services and their employer. The employer's contribution varied based on the labor contract, region, and company insurance. For white-collar workers, 80 % of their pre-tax salary was provided for three weeks, averaging SEK 31 920 (USD 3129, Euro 2705, Yen 529 872) (SEK 33 600 (USD 3294, Euro 2847, Yen 557 760) for men, SEK 30 240 (USD 2965, Euro 2562, Yen 501 984) for women); this amounted to SEK 2,257,920 (USD 221 365, Euro 191 349, Yen 37481472) (SEK 806 400 (USD 79 059, Euro 68 339, Yen 13 386 240) for men, SEK 1,451,520 (USD 142 306, Euro 123 010, Yen 24 095 232) for women). For blue-collar workers, 80 % of their pre-tax salary was provided for three months; this amounted to SEK 12,206,880 (USD 1 196 753, Euro 1 034 481, Yen 202 634 208) (SEK 5,947,200 (USD 583 059, Euro 504 000, Yen 98 723 520) for men, SEK 6,259,680 (USD 61 341, Euro 530 481, Yen 103 910 688) for women).

The total estimated cost of maltreated and misdiagnosed finger fractures from 2011 to 2021 amounted to SEK 25 557 200 (USD 2 505 608, Euro 2 165 864, Yen 424 249 520). (SEK 11,092,400 (USD 1 087 490, Euro 940 034, Yen 184 133 940) in direct cost and SEK 14,464,800 (USD 1 418 118, Euro 1 225 831, Yen 240 115 680) in indirect cost). The average cost per case was SEK 66 555 (USD 6525, Euro 5640, Yen 1 104 813) (SEK 72 430 (USD 7101, Euro 6138, Yen 1 202 338) for men, SEK 62 358 (USD 6114, Euro 5284, Yen 1 035 149) for women).

## 4 Discussion

The main finding of this study was the occurrence of 384 reported and accepted cases of maltreated and misdiagnosed finger fractures between 2011 and 2021. No significant trend was shown over time, although it should be noted that a possible lag time between injury and filing a claim may introduce some uncertainty. A similar study showed a positive trend towards an increase in total claims made to Löf of 6 % annually.^14^ In contrast, studies have also shown a statistically significant decrease in claims made to Löf regarding scaphoid fractures and wrist ligament injuries.^15^ Fractures occurred in people of all ages, and in this study, there was a higher number of maltreated and misdiagnosed fractures in women. In total, 75 % of the fractures occurred in people 18–65 years of age. The highest incidence of healthcare-related injuries regarding finger fractures occurred in the emergency care department, amounting to 40 % of all reported and accepted cases. Most of these cases were due to maltreatment (69 %, *n* = 105), leading to malunion of the fracture following non-operative treatment. Some of these patients should have been referred to a specialist for a second opinion regarding the fracture. That they were not referred could have resulted from a lack of knowledge on the part of the physicians in the emergency department or from the high workload at the emergency care facility. In addition, patients may have been referred but may for patient-related reasons not followed-up.^16^ Increasing workload and junior physicians in the emergency care department could be factors contributing to a higher frequency of maltreatments.

Healthcare-related injuries in the primary care department were largely due to misdiagnosis of the fracture, as has also been indicated by other studies in Sweden showing that the most common missed fractures in primary care are in the hand.^9^ This could partly be because most primary care units do not have X-ray devices, and fractures are easily interpreted as distorsions.

The second highest incidence of healthcare-related injuries regarding finger fractures occurred in specialist care, amounting to 36 % of all reported and accepted cases. Almost all of these were due to maltreatment (97 %, *n* = 134) after surgical intervention leading to malunion of the fracture. The high percentages of healthcare-related injuries could be due to the nature of the fracture, with more complicated fractures requiring surgical intervention, possibly due to rotational malalignment or too large angular malalignment. Surgical intervention can be challenging due to the complex anatomy and the need to address the joint, bone, and soft tissue simultaneously.^10^ In this study, we found that patients who had surgical treatment as the initial treatment were more likely to require corrective surgery. Some patients with healthcare-related injuries in specialist care had very complex fractures that required surgery. It could be that some patients had complications that were unavoidable due to the nature of the complicated fracture.

Of the maltreated and misdiagnosed patients in the specialist department who received surgical treatment, 78 % had K-wire fixation. Studies have shown that K-wire fixation produces good results with few complications such as scarring and tendon irritation, as well as being the most common surgical technique for finger fractures.^17^^,^^18^ Because of the nature of this study examining only maltreated and misdiagnosed cases, it is difficult to say for certain whether the present results match the real correlations among all treated finger fractures and the maltreated fractures. K-wire fixation could have been associated with more complications because of the treatment method; however, we speculated that this explanation was unlikely because of multiple studies showing it to be a method with few complications. We suggest that the explanation is that K-wire fixation was the most common treatment for all treated finger fractures, so it was not unlikely for some patients to have complications.

Comparing the two largest groups, i.e., K-wire and screw fixation, we could see that the number of re-operations was similar, at 46 % for K-wire and 50 % for screw fixation. We found no correlation between surgical technique and what finger was maltreated. There was no clear correlation between surgical method and complication rates, with stiffness after K-wire fixation amounting to 69 % and after screw fixation to 41 %. K-wire fixation is less stable than screw fixation, which limits the early mobilization and could be a factor contributing to the higher number of stiffness complaints with K-wire than screw fixation.^19^ Cases treated with K-wires may have differed in fracture pattern compared to cases treated with screws. In addition, differences in fracture complexity may have led to treatment selection and influencing outcome. Fracture complexity, rather than surgical method, may influence outcome variation. Patients who had non-operative treatment had residual stiffness more often than did patients who had surgery. We speculate that patients who had surgery received follow-up examinations and physical therapy, whereas patients who had non-operative treatment might not have had the same follow-up regime.

Since 2011, the Swedish National Fracture Registry (SFR) has collected population-level data on fractures.^20^ The total number of finger fractures reported to SFR between 2012 and 2019 was 21 341 fractures.^3^ During the same period, Löf accepted 289 cases of maltreated and misdiagnosed finger fractures, representing 1 % of finger fractures. This could seem low, but it is important to be aware that not all fractures may have been correctly reported to SFR by the clinics. Not all patients report maltreatments either due to unawareness or a decision not to file a claim. Therefore, the actual percentage of maltreated and misdiagnosed fractures could be higher and the sample biased. If the 1 % figure for such healthcare-related injuries is accurate, it would mean that 99 % of finger fractures were treated successfully, which would indicate that maltreated and misdiagnosed finger fractures are a relatively minor problem in Swedish healthcare. This is an approximation, as some underreporting and non-inclusion bias are likely in this study.

The most common maltreated and misdiagnosed finger fractured was the fifth finger, with a study showing that fractures often occurred on the outer edges of the hand, due their anatomically exposed location.^21^ This could be explained by the fifth finger being fractured more often, regardless of its being maltreated or not, so the higher number of maltreated fractures on the fifth finger simply reflects the normal distribution of finger fractures.

Subsequent medical invalidity after a maltreated finger fracture was high (70 %, *n* = 270), although the average degree of medical invalidity was relatively low at 2 %. Medical invalidity is calculated based on a set framework. Examples of a two per cent invalidity are: pulp-to-palm diastasis with clenched fist of 3 cm with one finger or fixation of digitus II in the proximal interphalangeal joint of 10–50°. The hand is essential in many professions, and even a slight invalidity can affect a patient's ability to work.^22^ For white-collar workers, a 2 % invalidity might not have a significant impact, but for blue-collar workers who rely on fine motor skills, it can have a substantial impact on their ability to work and to maintain their job.

The estimated sum of all costs (SEK 25 557 200 (USD 2 505 608, Euro 2 165 864, Yen 424 249 520) might not have seemed like a lot during the study period. However, the hand is an important tool and the impact of any hand injury on daily life is great, regardless of the cost.^21^^,^^23^ The quantified costs are not the only ones to consider. Two other avoidable costs were those of re-operations and the additional healthcare resources required due to maltreatment or misdiagnosed finger fractures. These costs are difficult to quantify and are not explored here, but highlight the potential economic impact. Additionally, healthcare-related injuries could negatively affect the patient's private finances, as sick pay, even if received, is typically less than regular income. This results in both higher costs for society as well as a negative impact on the patient's private finances.

This study had some limitations. First, only patient-reported maltreated and misdiagnosed finger fractures were included, potentially overlooking unreported cases. As a result, the number of misdiagnosed and maltreated fractures at the three main injury locations could be inaccurate, especially if reporting tendencies differ. In addition, the decision for an injury to be deemed avoidable is legal rather than a clinical diagnosis of maltreatment. For example, misdiagnosed cases could be underreported, as some patients may not seek further medical attention after a diagnosis of distorsion, leaving fractures undiagnosed.

Second, there is a risk of selection bias due to the filtering based on ICD-10-SE codes. If the injury was mislabeled in the database, it was excluded from the results. It is also important to note that only one investigator reviewed the data, resulting in a risk of misinterpretation.

Third, the Mann–Kendall test is designed to detect monotonic trends. The data analyzed in this study do not follow a monotonic trend, but instead vary over time. Therefore, the test is not fully applicable, although no significant trend over time could be seen.

Fourth, the approximation of indirect costs is uncertain, as it relies on recommended rather than actual sick leave which may reduce the cost validity. The Löf register does not systematically track sick-leave data, leading to potential overestimation in the case of patients who returned to work earlier than recommended. At the same time, some patients with multiple surgeries and long rehabilitations likely had extended sick leave, exceeding recommendations. A likely result is a total underestimation of the indirect costs. Including actual sick-leave data would improve future studies.

Fifth, patients who did not want their data used according to the opt-out principle may have differed systematically from patients who were included in the register.

This study had several strengths, such as the use of a national registry, a broad search strategy, and a systematic review of all included cases. The limitations likely led to underestimations, meaning that the findings would probably be more substantial if all cases were reported.

## 5 Conclusion

Finger fractures affect people of all ages and can lead to significant socioeconomic impacts and medical invalidity. Total direct and indirect cost amounted to SEK 25.5 million (USD 2.5 million, Euro 2.2 million, Yen 423 million) during the studied period. Maltreated fractures mainly occur in the emergency department, due to malunion after immobilization, and in specialist care, due to malunion after surgery. Misdiagnosed fractures are more common in primary care, often due to inadequate examinations and lack of X-ray examinations. We found no significant correlation between surgical technique and healthcare-related injuries.

To reduce the incidence of these injuries, a suggestion for claims prevention would be improved physician education, the more liberal use of X-rays, and seeking specialist consultations in uncertain cases. Additionally, evaluating surgical techniques and optimizing postoperative care could further reduce these injuries.

## Ethics

The study obtained ethical approval from the Swedish Ethical Review Authority (Dnr 2022-07279-01). Individual informed consent was not sought for the present study, in accordance with Swedish law. Informed consent was given by the patients to the Löf-register at the time of filing a complaint.

## Funding

This study was funded by a grant from Örebro County Council (ALF project, grant number: OLL-1010486). The funding bodies had no part in the design of the study, collection, analysis and interpretation of data, or in the writing of the manuscript.

## Declaration of competing interest

The authors declare that they have no known competing financial interests or personal relationships that could have appeared to influence the work reported in this paper.
